# Does Arterial Blood Gas (ABG) Provide a Safety Net for Extubation in Surgical Patients?

**DOI:** 10.7759/cureus.33561

**Published:** 2023-01-09

**Authors:** Amarjyoti Hazarika, Karan Singla, Gautham Patel, Varun Mahajan, Shubhkarman Kahlon, Shyam Meena

**Affiliations:** 1 Anaesthesia and Intensive Care, Postgraduate Institute of Medical Education and Research, Chandigarh, IND

**Keywords:** bicarbonate, reintubation, blood gas analysis, weaning, extubation, anesthesia

## Abstract

Background

Extubation has always been a critical aspect of anaesthesia. Guidelines and recommendations are in place for achieving successful extubation, but the risk of failure always persists. Through this study, we assess whether arterial blood gas (ABG) values taken intraoperatively help predict extubation success in the operation theatre.

Materials and methods

This was a prospective observational study for one year of extubated patients whose blood gas values were not within the normal range. The patients of age 18 years and above undergoing high-risk elective and emergency surgeries where at least one intraoperative arterial blood sample was taken for blood gas analysis were included. Apart from parameters of ABG demographic data, urgency and duration of surgery, blood loss, urine output, use of intraoperative fluid(s), and blood product(s) were also observed.

Results

Of 578 patients enrolled, 116 patients were extubated based on the predefined extubation criteria. Of these, 24 patients were reintubated within 24 hours. ABG parameters such as partial pressure of arterial oxygen (PaO_2_) and serum HCO_3- _levels were significantly lower in the reintubated patients compared to non-reintubated patients (p-values of 0.045 and 0.003, respectively).

Conclusion

This study showed that the PaO_2_ <100 mm Hg or ratio of arterial oxygen partial pressure to fractional inspired oxygen (P/F ratio) of less than 200 and an HCO_3-_ value of less than 18 are plausible ABG parameters to decide extubation in post-surgery patients in OT. PaCO_2_, base deficit, and lactate were less reliable parameters for planning extubation.

## Introduction

Extubation has always been a perilous exercise in the practice of anaesthesia. There have been guidelines and recommendations for achieving successful extubation, but the risk of failure always persists, especially in post-high-risk surgeries. Guidelines in place consider apparent clinical features. In high-risk surgery, patients' physiology is subjected to a wide variation of hemodynamic changes owing to fluid shift, blood loss, and above all, the nature of the underlying pathology being treated. This invariably affects variables like acid-base balance and blood gas values, which also significantly affect the favorable outcome of extubation. These variables fall under the ambit of blood-gas analysis; most commonly, arterial blood samples are used for this analysis. They represent a summary of many events that include cellular metabolism, muscle activity, oxygen consumption, carbon dioxide production, cardiac function, tissue perfusion, ventilation, and perfusion. ABG analysis has been in practice as one of the essential factors for weaning and extubating patient from mechanical ventilation in intensive care settings [1]. ABG values were also used to gradually reduce intermittent mandatory ventilation in post-surgery patients [2]. However, there is no agreement on the use of ABG values as a factor in assessing extubation decisions in operation theatre (OT). Nonetheless, many centers measure ABGs and use their values in conjunction with clinical data to make extubation decisions. Through this study, we want to assess whether abnormal intraoperative ABG values can solely be used in reversing extubation decisions even if set extubation guidelines are fulfilled. We hypothesized that ABG values during surgery are not a predictive factor for deciding on extubation. The primary objective of this prospective observational study was to assess ABG values as a predictor for deciding on extubation in OT. The secondary objective was to assess intraoperative parameters, i.e., the urgency of surgery blood loss, urine output, use of intraoperative fluid(s), and blood product(s) as factors in planning extubation after surgery in OT.

## Materials and methods

This was a prospective observational study of one year for patients extubated after high-risk surgery inside OT whose ABG values were not within the normal range. Approval from the Institutional Ethics Committee (NK/5242/Study/775) and submission in the clinical trials registry India (CTRI) REF/2022/07/056830) were taken for conducting this study. Informed consent from the patients or relatives was also taken. The study was done in accordance with the Ethical Principles for Medical Research Involving Human Subjects, outlined in the Helsinki Declaration of 1975 (revised 2013). The patients were managed and treated as per the standard departmental anaesthesia protocol. Included in the study were postoperative patients of age 18 years and above of either sex who were extubated in OT after high-risk elective or emergency surgery following fulfillment of predefined extubation criteria (Table 1) where at least one arterial blood sample was taken intraoperatively for ABG analysis. Postoperative patients with known chronic renal disease, renal tubular acidosis, sepsis, chronic obstructive pulmonary disease (COPD), traumatic brain injury, hyperventilation syndrome, polytrauma, pregnancy, undergoing cardiac surgery extubated in OT, and nonconsenting patients were excluded from the study.

**Table 1:** Predefined extubation criteria to be fulfilled before making the decision to extubate. Source: [3,4] RR: Respiratory rate; TOF ratio: Train-of-four ratio.

Adequate Oxygenation	SpO2 > 95%
Adequate Ventilation	Tidal volume > 6 ml/kg, spontaneous RR > 7 bpm, but less than 30 bpm, end-tidal carbon dioxide (ETCO2) < 50 mmHg
Hemodynamically Stable	Heart rate >50 bpm and <150 bpm, systolic blood pressure (SBP) >90 mmHg, diastolic blood pressure (DBP) >60 mmHg
Adequate Reversal of Muscle Relaxation	TOF ratio >0.9, sustained 5-second head lift or hand grasp
Neurologically Intact	Follows verbal commands, intact cough/gag reflex
Normovolemia	Central venous pressure (CVP) >8 cm, H2O pulse pressure variation <8%
Normal Electrolytes	Serum sodium - 135 mEq to 145 mEq/dl, serum potassium - 3.5-4.5 mEq/dl, serum calcium - 8-1.1 mEq/dl
Normothermic	Temperature >35.5˚ Celsius and <38º Celsius
No Aspiration Risk	
Absence of Excess Secretions	
No Airway Edema	Cuff leak volume of more than 110 ml or ~20% of tidal volume

Reintubation was defined as endotracheal intubation within 24 hours post-extubation. The parameters observed for ABG analysis were pH, PaO_2_, PaO_2_/FiO_2_ (P/F ratio), PaCO_2_, HCO_3-_, and base difference (Table 2).

**Table 2:** Shows the arterial blood gas (ABG) values used in the study for reference, intraoperative parameters observed, and the type of surgery. PRBC: Packed red blood cells; FFP: Fresh frozen plasma; GI: Gastrointestinal.

Arterial blood gas	pH-7.35-7.45
	PaO_2_ > 60 mmHg
	PaCO_2_- 35-45mmHg
	HCO_3_^-^- 22-28 meq/L
	Base excess ­2 to +2 mmol/L
Intraoperative parameters	Age
	Urgency of surgery (elective, emergency)
	Duration
	Blood loss
	Urine output
	Fluid (normal saline, Ringer's lactate, colloid)
	Blood product (PRBC, FFP, platelet concentrate, cryoprecipitate)
Type of Surgery (High Risk )	Urology (radical prostatectomy, radical cystectomy with and without conduit)
	Vascular (abdominal aortic aneurysm repair, iliac artery repair)
	GI (laparotomy, hepatectomy, Whipple’s procedure)
	Gynaecological (total abdominal hysterectomy, laparotomy)
	Orthopaedic (joint replacement surgery requiring general anaesthesia)

The anaesthesia provider took the arterial blood samples from one of the accessible arteries, e.g., radial artery, dorsalis pedis artery, or posterior tibial artery, in a heparinized syringe. The samples were kept on an ice pack and analyzed within five minutes of withdrawing the sample. The potentiometry and amperometry technique was used for the analysis of the arterial blood samples. All was done in a situation where more than one sample was taken for analysis; the last sample before the end of surgery or before deciding on extubation was included in the study. The ABG values not within the range were taken as abnormal.

The data were analyzed using SPSS software version 21 for Microsoft Windows (SPSS Inc., Chicago, IL, USA). Nonparametric simple descriptors (median, range, and interquartile range) were used to calculate continuous numeric data (e.g., age, duration, etc.). For checking the association between the variables, Fisher's exact test was used for the categorical data (e.g., blood loss, urine output, etc.), and Mann- Whitney U test was used for continuous variables (e.g., ABG values). Multivariate analyses were used to determine possible relationships between the factors and outcomes. A p-value of <0.05 was considered to be statistically significant.

## Results

**Figure 1:**
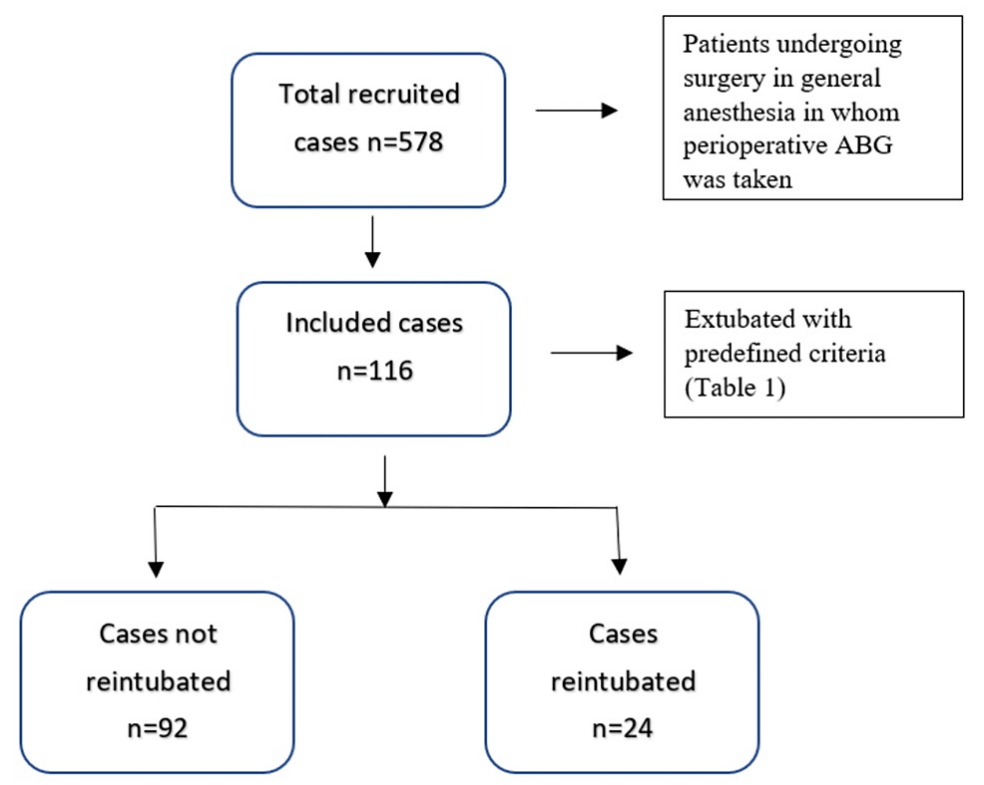
Consolidated standards of reporting trials. n: Number of participants; ABG: Arterial blood gas.

Of 578 patients enrolled as per inclusion criteria, 116 were extubated based on the predefined extubation criteria (Figure 1).

Of these, 24 patients were reintubated within 24 hours (Table 3). There was no difference between the groups in elective, emergency surgeries, and total blood loss. Median urine output was less in the reintubated group (350 ml vs. 400 ml, p-value: 0.035). The type and amount of intraoperative fluid and use of blood products also showed no significant difference between the groups.

**Table 3:** Comparing demographic data, urgency and duration of surgery, blood loss, urine output, use of intraoperative fluid(s), and blood product(s). NS: Normal saline; RL: Ringer lactate; PRBC: Packed red blood cell; FFP: Fresh frozen plasma.

	Reintubated (n-24)	Not reintubated (n-92)	P-value
Age	53.5 (41,63)	45.5(32.25,57)	0.101
Duration (hours)	3 (2.62,4.37)	4 (3,5)	0.033
Elective (n-59), emergency (n-57)	8 13	51 44	0.853
Blood loss (ml)	1250 (700,1800)	1400 (700,2000)	0.883
Urine output (ml)	350 (300,400)	400 (300,500)	0.035
Fluid (L)			
NS	2.75 (2,3.75)	3 (2,4)	0.883
RL	1 (0.5,1)	1 (0.5,2)	0.131
Colloids	0.75 (0.5,1)	0.25 (0,1)	0.223
Blood products (Units)			
PRBC	66	270	0.789
FFP	29	114	1.00
Platelets	31	120	1.00
Cryoprecipitate	2	2	1.00

ABG parameters PaO_2_ and HCO_3-_ were significantly lower in the reintubated patients as compared to not reintubated patients (p-values of 0.045 and 0.003, respectively). Although the difference in the pH values between the two groups is statistically different, the actual difference is small (Table 4). Eleven patients in the not reintubation group and eight patients in the reintubation group had pH<7.3, with a greater proportion in the latter (Table 4).

**Table 4:** ABG values of reintubated and not reintubated patients. *Mann-Whitney U test. The p-values reported in bold are significant. n: Number of patients; IQ: Interquartile range; pH: Potential of hydrogen; PaO_2_: Partial pressure of arterial oxygen in mmHg; PaCO_2_: Partial pressure of arterial carbon dioxide in mmHg; HCO_3_^-^: Bicarbonate concentrado in mmoL/L; BE: Base excess; ABG: Arterial blood gas.

ABG values	Reintubated (n-24) Median (IQ)	Not reintubated (n-92) Median (IQ)	P-value*
pH	7.31 (7.29, 7.32)	7.32 (7.3, 7.33)	0.028
PaO_2_(mm Hg)	193 (153.04, 201.5)	203 (162.5, 274.5)	0.045
PaCO_2 _(mm Hg)	32.80 (31.6, 34.85)	33.25 (31.92, 36)	0.217
HCO_3 _(mmol/L)	17.85 (16.7, 18.67)	18.70 (17.6, 20)	0.003
BE (mmol/L)	-7.35 (-8.4, -5.22)	-6.4 (-7.9, -4.95)	0.100
Lactate (mmol/L)	2.55 (2.2, 3.4)	2.70 (2.2, 3.6)	0.981

**Figure 2:**
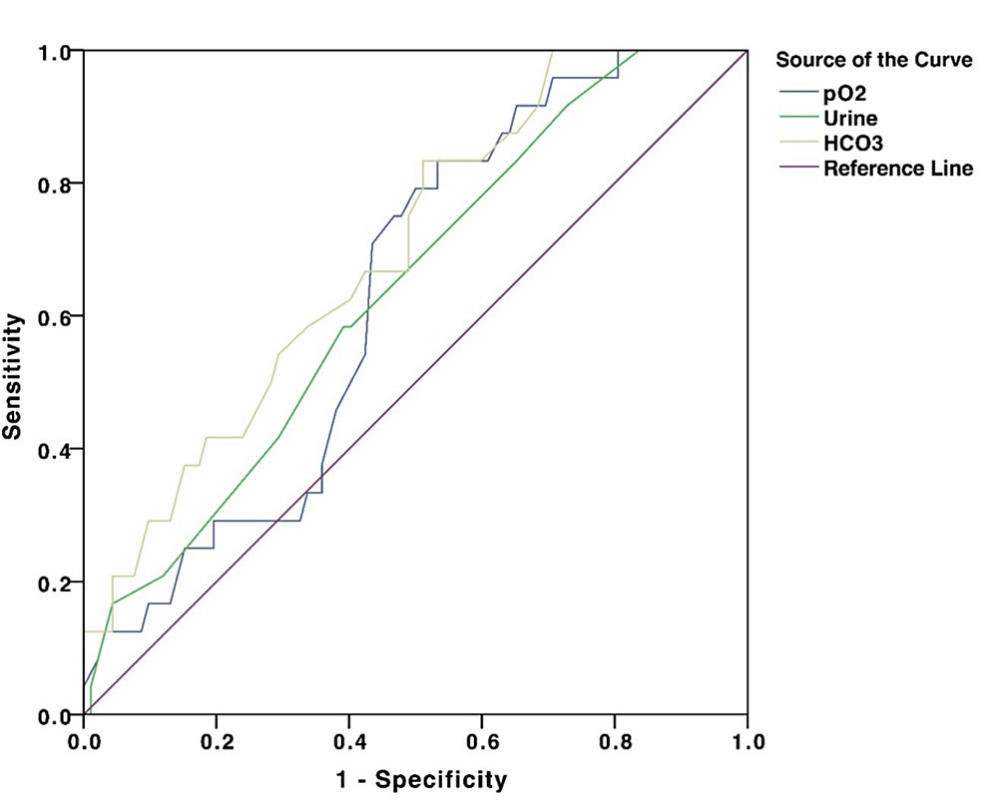
The ROC curve of PaO2, urine output and HCO3- values. The AUCs were 0.633, 0.638, and 0.696, respectively. ROC: Receiver operating characteristics; AUC: Area under the curve; PaO_2_: Partial pressure of arterial oxygen in mmHg: HCO_3_^-^: Bicarbonate.

However, PaCO_^2^_, base deficit, and lactate had no significant difference between the groups. In the receiver operating characteristics (ROC) analysis, the area under the curve (AUC) of PaO_2_ was 0.633 (95% CI: 0.523-0.743, p=0.045; urine output was 0.638 [95% CI: 0.522-0.745, p=0.038]; and for HCO_3-_ was 0.696 [95% CI: 0.586-0.806, p=0.003]) (Figure 2). A PaO_2_ of <100 mmHg had a sensitivity of 70.8% and specificity of 56.5%, urine output of <387.5 ml had a sensitivity of 58.3% and specificity of 60%, and a serum HCO_3-_ <18.65 mol/L had a sensitivity of 75% and specificity of 51%. ROC analysis is not reported for pH because the difference in absolute values between the two groups was too small to get a clinically significant cut-off. 

## Discussion

This study showed that PaO_2_ ratio and serum HCO_3_^- ^values are plausible ABG parameters to decide on extubation in post-surgery patients undergoing high-risk surgeries in OT. These values, together with intraoperative urine volume, may be useful parameters in deciding extubation success or failure in this group of patients when predefined extubation criteria are fulfilled. PaCO_2_, base deficit, and lactate were less reliable parameters for planning extubation.

It is a proverb that intubation denotes skill and extubation is an art. With the growth of science and point-of-care testing like arterial gas analysis, quantifiable data can be made available that helps in planning extubation with almost accurate predictions. Planned extubation is generally considered safe and practical. However, reintubation after planned extubation is not uncommon. The rate of reintubation varies from 0.17 to 0.27% [3-4]. Literature has stressed the importance of extubation criteria and the need to follow them to achieve that goal. Extubation criteria in the operating room based on subjective parameters like sustained head raise have their fallacies. ABG values have been used to guide extubation decisions in ICU settings, but OT studies validating its usefulness are scarce.

Several studies have underlined the importance of a P/F ratio >200 as a benchmark for the increase in extubation success rate [5-6]. This study found that the PaO_2_ <100 (on FiO_2 _of 0.5 and hence P/F ratio of 200 mm Hg) is associated with unfavorable extubation outcomes. However, there were 40 (out of a total of 90 ) patients who had a PaO_2_ less than 100 mm Hg on FiO_2 _of 0.5 and did not require reintubation. This may be ascribed to adherence to extubation criteria before deciding on it. The majority of the literature on the P/F ratio is in ICU settings where patients are put on mechanical ventilation due to respiratory insufficiency. In contrast, in our study, ventilatory necessity is due to operative requirements. Moreover, in our study, the ABGs were analyzed intraoperatively using FiO_2_ of 0.5 in contrast to ICU settings where variable and decreasing FiO_2_ are employed before extubation readiness. Studies have often quoted HCO_3_^- ^levels less than 18 mmol/L to be associated with extubation failure [7]. Similarly, low HCO_3_^-^ levels have been related to an increase in the reintubation rate [8]. It is also an independent factor in predicting extubation outcomes [9]. In our study, HCO_3_^-^ values less than 18 mmol/L were a predictive factor for reintubation. Low serum HCO_3_^-^ level is an indicator of metabolic acidosis, which is due to inadequate tissue perfusion (generalized or localized to some organ systems) leading to anaerobic metabolism. This may happen even in presence of objective hemodynamic stability and ventilation. So, even if another parameter normalizes and serum HCO_3_^- ^level^ ^is_ _still low, then one must consider its importance during extubation as its correction is slow due to delayed renal response. Our study found pH to be significantly different between the groups. However, a clinically valuable cut-off could not be achieved, unlike previous studies that showed pH<7.3 is associated with extubation failure [7]. We could not show pH as a factor in deciding extubation readiness as none of the patients had a pH value <7.3. Arterial blood pH is a marker of the different physiological mechanisms in place to keep the body and its organs functioning in times of stress and strain. Hence pH value is essential before planning extubation.

In our study, the incidence of reintubation was more with an increase in operating time (median 4 hours vs. 3 hours) and low urine output. Our findings were similar to a study where an operative time of more than three hours was an independent risk factor for reintubation [10]. Previous studies also showed increased reintubation risk with longer operative time [11] and low urine output. This study found low urine volume as one of the predictive factors for reintubation. Urine volume is an indicator of renal physiology, and a decreased amount might indicate renal insufficiency leading to reintubation [12]. No previous studies have considered intra-operative urine volume in extubation criteria. Our study found that the nature of the surgery (whether elective or emergency), amount of surgical blood loss, intraoperative fluid, or use of blood products had no bearing on the reintubation rate. This was in contrast to studies that associated these parameters with an increased risk of reintubation [13-16]. This may be due to the present study adhering to an inclusion criterion (exclusion of patients with COPD, renal disease, sepsis, etc.) with a set of predefined parameters that must be confirmed before extubation.

Although ABG values may impact the extubation decisions, they did not change the management of patients very much when added to the clinical assessment [17]. Accordingly, there is no consensus regarding whether ABGs are routinely necessary for extubation readiness, even in ICU settings. Our study also found that all parameters of ABG may not be the sole benchmark for making decisions involving extubation when bio-clinical criteria are met. Practitioners may not measure ABGs for extubation decisions if it does not involve high-risk surgeries. As such, substantial heterogeneity of practice prevails regarding the reliability of ABG for predicting extubation outcomes. Though this is a one-of-a-kind protocolized study, it has its limitations. This being an observational study with a small sample size, the type of surgeries taken in the study did not belong to low-risk surgery, a common subspecialty, nor was it performed by a single surgeon. Hence skill of the surgeon and the type of surgery may definitely have a bearing on the outcome. Also, this study reflects the outcome data at one center, and there was no control group with which outcomes could be compared. Even though protocolized, the timing of ABG before the end of surgery was not timed precisely across the participants. Our study excluded patients with COPD, renal disease, and sepsis who were at high risk for extubation failure.

## Conclusions

In conclusion, the current study establishes that along with predefined extubation criteria, a PaO_2_ <100 mm Hg or P/F ratio of <200 mm Hg (in our study, the ABGs were analyzed intraoperatively using FiO_2_ of 0.5), serum HCO3- values <18 mmol/L and low intraoperative urine volume in the reintubated group are plausible ABG parameters to aid in decision making regarding extubation in post-surgical patients undergoing high-risk procedures. The incidence of reintubation was also more with an increase in operating time. Hence, extubation decisions in patients fulfilling these criteria may be deferred and probably mechanically ventilated till these parameters are correct. According to our study, other parameters on ABG, like pH, PaCO_2_, base deficit, and lactate, were less reliable in extubation decisions and warrant further investigations.

## References

[1] Johnson RG, Arozullah AM, Neumayer L, Henderson WG, Hosokawa P, Khuri SF (2007). Multivariable predictors of postoperative respiratory failure after general and vascular surgery: results from the patient safety in surgery study. J Am Coll Surg.

[2] de Lassence A, Alberti C, Azoulay E (2002). Impact of unplanned extubation and reintubation after weaning on nosocomial pneumonia risk in the intensive care unit: a prospective multicenter study. Anesthesiology.

[3] Lee PJ, MacLennan A, Naughton NN, O'Reilly M (2003). An analysis of reintubations from a quality assurance database of 152,000 cases. J Clin Anesth.

[4] Chinachoti T, Chau-in W, Suraseranivongse S, Kitsampanwong W, Kongrit P (2005). Postoperative reintubation after planned extubation in Thai Anesthesia Incidents Study (THAI Study). J Med Assoc Thai.

[5] Khamiees M, Raju P, DeGirolamo A, Amoateng-Adjepong Y, Manthous CA (2001). Predictors of extubation outcome in patients who have successfully completed a spontaneous breathing trial. Chest.

[6] Namen AM, Ely EW, Tatter SB (2001). Predictors of successful extubation in neurosurgical patients. Am J Respir Crit Care Med.

[7] Wang SH, Liou JY, Chen CY, Chou HC, Hsieh WS, Tsao PN (2017). Risk factors for extubation failure in extremely low birth weight infants. Pediatr Neonatol.

[8] Moura JC, Gianfrancesco L, Souza TH, Hortencio TD, Nogueira RJ (2021). Extubation in the pediatric intensive care unit: predictive methods. An integrative literature review. Rev Bras Ter Intensiva.

[9] Lin C, Gong B, Li Y (2018). Before extubation blood gas factors analysis of postoperative early withdraw ventilator influencing in in-fants with congenital heart diseases. J Pract Med.

[10] Legrand M, Payen D (2011). Understanding urine output in critically ill patients. Ann Intensive Care.

[11] Arozullah AM, Daley J, Henderson WG, Khuri SF (2000). Multifactorial risk index for predicting postoperative respiratory failure in men after major noncardiac surgery. The National Veterans Administration Surgical Quality Improvement Program. Ann Surg.

[12] Kim M, Rhim SC, Roh SW, Jeon SR (2018). Analysis of the risk factors associated with prolonged intubation or reintubation after anterior cervical spine surgery. J Korean Med Sci.

[13] Wang K, Yin Y (2017). Risk factors and prognosis of reintubation following surgeries under general anesthesia. Zhongguo Yi Xue Ke Xue Yuan Xue Bao.

[14] Ghosh S, Chawla A, Mishra K, Jhalani R, Salhotra R, Singh A (2018). Cumulative fluid balance and outcome of extubation: a prospective observational study from a general intensive care unit. Indian J Crit Care Med.

[15] Kipps AK, Wypij D, Thiagarajan RR, Bacha EA, Newburger JW (2011). Blood transfusion is associated with prolonged duration of mechanical ventilation in infants undergoing reparative cardiac surgery. Pediatr Crit Care Med.

[16] Salam A, Smina M, Gada P, Tilluckdharry L, Upadya A, Amoateng-Adjepong Y, Manthous CA (2003). The effect of arterial blood gas values on extubation decisions. Respir Care.

[17] Soo Hoo GW, Park L (2002). Variations in the measurement of weaning parameters: a survey of respiratory therapists. Chest.

